# Supplementary material for: A CRISPR/Cas9-induced restoration of bioluminescence reporter system for single-cell gene expression analysis in plants
Source: Sci Rep. 2025 Nov 21;15:41271. doi: 10.1038/s41598-025-25062-9 (PMC12638942; doi:10.1038/s41598-025-25062-9)
Supplement: Supplementary file 1 — Supplementary Material 1 [file 41598_2025_25062_MOESM1_ESM.pdf]

Supplementary information of "A CRISPR/Cas9-induced restoration of bioluminescence reporter system for single-cell gene expression analysis in plants".

Authors

Ryohei Ueno[1], Shogo Ito[1] & Tokitaka Oyama[1*]

Affiliation

[1]Department of Botany, Graduate School of Science, Kyoto University, Kitashirakawa-Oiwake-cho, Sakyo-ku, Kyoto, 606-8502, Japan

*Corresponding Author

Tokitaka Oyama, Ph. D.

Phone: +81 75 753 4135

Fax: +81 75 753 4137

Email: oyama.tokitaka.8w@kyoto-u.ac.jp

This file includes:

Supplementary Table S1–3 (list, page 2; tables, pages 4–6)
Supplementary Fig. S1–11 (list, page 2; figures, pages 7–17)
List of Supplementary Spreadsheet S1–2 (page 3)
List of Supplementary Video S1–5 (page 3)

Supplementary Table S1. The primers used for PCR amplification to construct *pENTR_LUC2Ins24bp/26bp*.

Supplementary Table S2. The primers used for PCR amplification to construct *pENTR_LUCX$_{AA}$Ins24bp/26bp* except for *pENTR_LUC2Ins24bp/26bp*.

Supplementary Table S3. Plasmid concentrations and amounts used for particle bombardment.

Supplementary Fig. S1. Bioluminescence intensities of duckweed cells transfected with *LUCX$_{AA}$Ins24bp*.

Supplementary Fig. S2. Bioluminescence spectra of *LUCX$_{AA}$Ins24bp*.

Supplementary Fig. S3. Bioluminescence in duckweed cells co-transfected with *LUC40Ins24bp* or *LUC40Ins26bp* and nonsense-*CRISPR/Cas9* constructs.

Supplementary Fig. S4. Effects of the *CRISPR/Cas9* constructs on bioluminescence rhythms of duckweed plants transiently transfected with *AtCCA1::LUC40Ins24bp* and *AtCCA1::LUC40Ins26bp* reporters.

Supplementary Fig. S5. mRNA expression levels of *LUC40Ins26bp* in T2 transgenic *Arabidopsis* plants carrying *CaMV35S::LUC40Ins26bp*.

Supplementary Fig. S6. Relationship between the ploidy profile of *Arabidopsis* leaf cells and the ratio of bioluminescent spots.

Supplementary Fig. S7. Cellular bioluminescence traces of restored-*LUC40Ins26bp* in transgenic plants (line *LUC40Ins26bp* #38-2).

Supplementary Fig. S8. *LU_UC* construct and its bioluminescence properties.

Supplementary Fig. S9. Comparison of cellular bioluminescence intensities between duckweed cells co-transfected with *LU_UC* and nonsense-*CRISPR/Cas9* or *CRISPR/Cas9* constructs.

Supplementary Fig. S10. Split *LUC40Ins24bp* constructs (*nLUC40* and *cLUC40*) and bioluminescence properties.

Supplementary Fig. S11. Sequence information of *pR4GWB501_CaMV35S::LUC40Ins26bp*.

Supplementary Spreadsheet S1. This Excel file contains a simulator that calculates the ratio of bioluminescence-restored cells to transfected cells from the probability of optimal recombination.

Supplementary Spreadsheet S2. This Excel file contains a simulator that calculates the proportion of cells carrying only one chromosome with the optimal recombination among all bioluminescence-restored cells from the probability of optimal recombination.

Supplementary Video S1. Time-course monitoring of bioluminescence of *LUC40Ins26bp* #28-3 leaves transfected with *CRISPR/Cas9* constructs (left) and of *Col-0* leaves transfected with *CaMV35S::LUC+* (right). Bioluminescence images were hourly captured under constant dark conditions. Time (h) after gene transfection is indicated. Exposure time: (left) 150 and (right) 60 s. The signal range was fixed to 1920–2200. Each bright-field image is shown at the beginning of the video. Bars: 10 mm.

Supplementary Video S2. Time-course monitoring of *LUC40Ins26bp* #28-3 leaves transfected with nonsense-*CRISPR/Cas9* constructs (left), *pENTR_AtU6-26::sgRNA-*$LUCX_{AA}$ (middle), and *pUC18_PcUBQ4-2::Cas9* (right). Bioluminescence images were hourly captured under constant dark conditions. Time (h) after gene transfection is indicated. Exposure time: 150s. The signal range was fixed to 1920–2200. Each bright-field image is shown at the beginning of the video. Bars: 10 mm.

Supplementary Video S3. Time-course close-up monitoring of bioluminescence of *LUC40Ins26bp* #28-3 leaves transfected with *CRISPR/Cas9* constructs (left) and of *Col-0* leaves transfected with *CaMV35S::LUC+* (right). Bioluminescence images were hourly captured under constant dark conditions. Time (h) after gene transfection is indicated. Arrows represent leaves of interest. Exposure time: (left) 150 and (right) 60 s. The signal range: (left) 1920–2500 and (right) 1920-15000. Each bright-field image is shown at the beginning of the video. Bars: 10 mm.

Supplementary Video S4. Time-course monitoring of bioluminescence of *LUC40Ins26bp* #28-3 leaves transfected with *CRISPR/Cas9* constructs for the single-cell analysis. Time 0 (h) indicates the start of the constant light. Exposure time: 150 s. The signal range: 1920–7000. Bright-field image is shown at the beginning of the video. Bar: 2 mm.

Supplementary Video S5. Time-course monitoring of bioluminescence of *LUC40Ins26bp* #38-2 leaves transfected with *CRISPR/Cas9* constructs for the single-cell analysis. Time 0 (h) indicates the start of the constant light. Exposure time: 150 s. The signal range: 1920–7000. Bright-field image is shown at the beginning of the video. Bar: 5 mm.

Supplementary Table S1. The primers used for PCR amplification to construct *pENTR_LUC2Ins24bp/26bp*

forward primers (from 5′ to 3′)	
LUC2Ins24bp	CTATGATTCCCAGCGGCACCGACGCCAAAAACATAAAGAAAGGC
LUC2Ins26bp	CTATGATTCCCAGCGGTTAACCGACGCCAAAAACATAAAGAAAGGC
reverse primer (from 5′ to 3′)	
LUC2Ins24bp/26bp	CCGCTGGGAATCATAGGTAGAACGACCATGGCGGCCGCGGAGCCTGC

Supplementary Table S2. The primers used for PCR amplification to construct *pENTR_LUCX$_{AA}$Ins24bp/26bp* except for *pENTR_LUC2Ins24bp/26bp*

reverse primers for the *5′-part-LUC* fragment (from 5′ to 3′)	
LUC40Ins24bp/26bp	CCGCTGGGAATCATAGGTAGAACTGTTCCAGGAACCAGGGCGT
LUC106Ins24bp/26bp	CCGCTGGGAATCATAGGTAGAACCGCGGGCGCAACTGCAACTC
LUC239Ins24bp/26bp	CCGCTGGGAATCATAGGTAGAACTAAAATCGCAGTATCCGGAA
LUC378Ins24bp/26bp	CCGCTGGGAATCATAGGTAGAACATCCAGATCCACAACCTTCG
LUC491Ins24bp/26bp	CCGCTGGGAATCATAGGTAGAACTCCGTGCTCCAAAACAACAA
forward primers for the *3′-part-LUC* fragment (from 5′ to 3′)	
LUC40Ins24bp	GTTCTACCTATGATTCCCAGCGGCATTGCTTTTACAGATGCACA
LUC40Ins26bp	GTTCTACCTATGATTCCCAGCGGTTAATTGCTTTTACAGATGCACA
LUC106Ins24bp	GTTCTACCTATGATTCCCAGCGGCAACGACATTTATAATGAACG
LUC106Ins26bp	GTTCTACCTATGATTCCCAGCGGTTAAACGACATTTATAATGAACG
LUC239Ins24bp	GTTCTACCTATGATTCCCAGCGGCAGTGTTGTTCCATTCCATCA
LUC239Ins26bp	GTTCTACCTATGATTCCCAGCGGTTAAGTGTTGTTCCATTCCATCA
LUC378Ins24bp	GTTCTACCTATGATTCCCAGCGGCACCGGGAAAACGCTGGGCGT
LUC378Ins26bp	GTTCTACCTATGATTCCCAGCGGTTAACCGGGAAAACGCTGGGCGT
LUC491Ins24bp	GTTCTACCTATGATTCCCAGCGGCAAGACGATGACGGAAAAAGA
LUC491Ins26bp	GTTCTACCTATGATTCCCAGCGGTTAAAGACGATGACGGAAAAAGA

Supplementary Table S3. Plasmid concentrations and amounts used for particle bombardment

plasmid components			concentration (ng/μL)	amount added (μL)
vector	promoter	gene		
pUC18	*CaMV35S*	*LUC2Ins24bp*	1023.5	2.0
		LUC40Ins24bp	989.7	2.0
		LUC106Ins24bp	998.0	2.0
		LUC239Ins24bp	999.9	2.0
		LUC378Ins24bp	982.0	2.0
		LUC491Ins24bp	1018.7	2.0
	CaMV35S	*LUC2Ins26bp*	236.1	8.5
		LUC40Ins26bp	997.4	2.0
		LUC106Ins26bp	366.9	5.5
		LUC239Ins26bp	355.4	5.6
		LUC378Ins26bp	327.8	6.1
		LUC491Ins26bp	335.8	6.0
	AtCCA1	*LUC40Ins24bp*	1008.2	2.0
		LUC40Ins26bp	977.1	2.0
pENTR	*AtU6-26*	*sgRNA_LUCX*$_{AA}$	998.9	1.5
		sgRNA	1000.9	1.5
pUC18	*PcUBQ4-2*	*Cas9*	1008.4	1.5

Supplementary Fig. S1

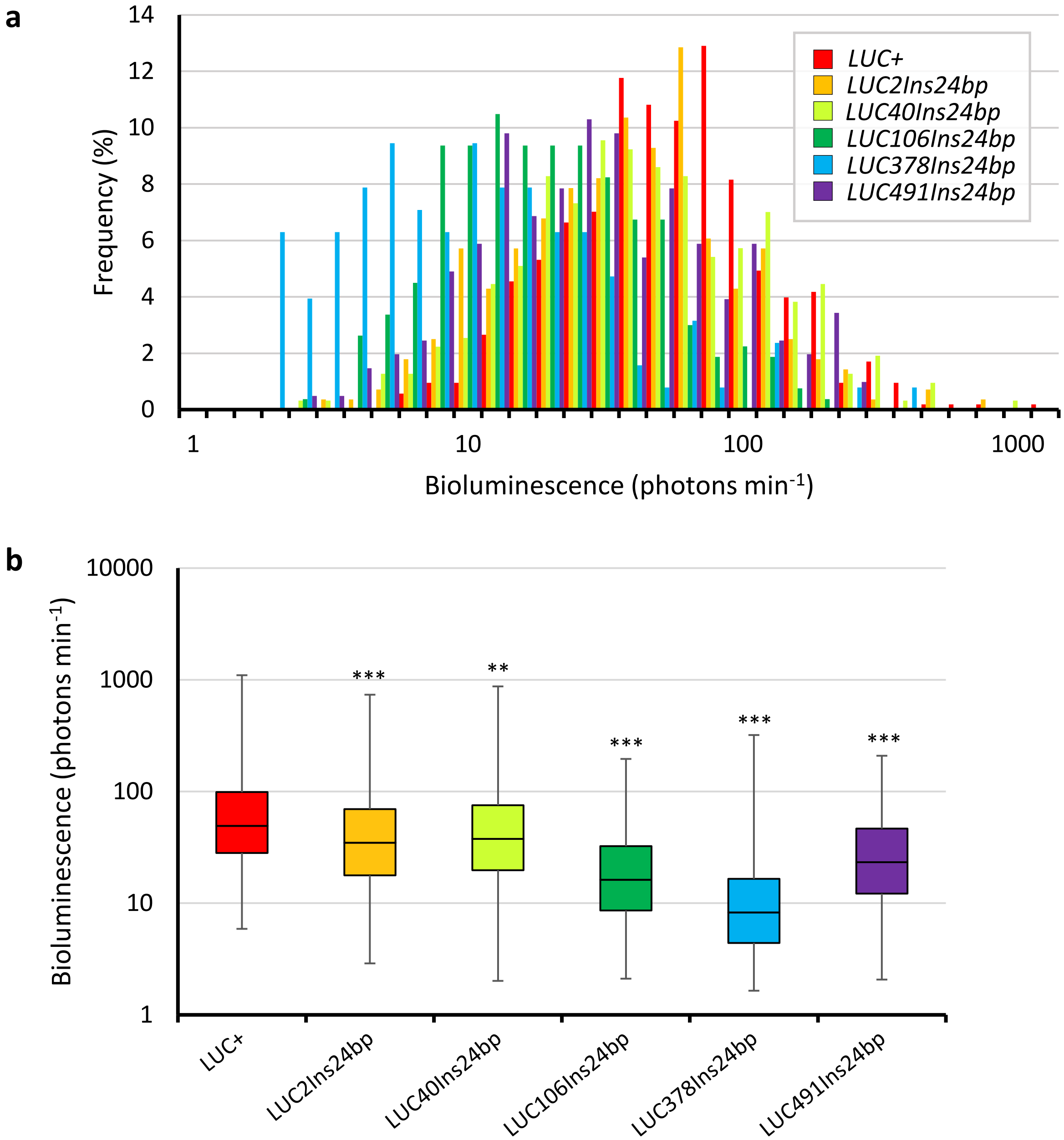

Bioluminescence intensities of duckweed cells transfected with *LUCX$_{AA}$Ins24bp*. (**a**) Frequency distribution (%) and (**b**) boxplot of cellular bioluminescence intensities for *CaMV35S::LUC+* (n = 526), *CaMV35S::LUC2Ins24bp* (n = 280), *CaMV35S::LUC40Ins24bp* (n = 314), *CaMV35S::LUC106Ins24bp* (n = 267), *CaMV35S::LUC378Ins24bp* (n = 127), and *CaMV35S::LUC491Ins24bp* (n = 204). Within each box for (**b**), the horizontal line represents the median value, the box extends from the 25th to the 75th percentile, and the vertical extended line ranges from the maximum to the minimum. The mean bioluminescence intensity for each construct is compared to that of *CaMV35S::LUC+* (Welch's two-tailed *t*-test; $^{**}p < 0.01$, $^{***}p < 0.001$). Twenty-five duckweed colonies were observed in one experiment.

Supplementary Fig. S2

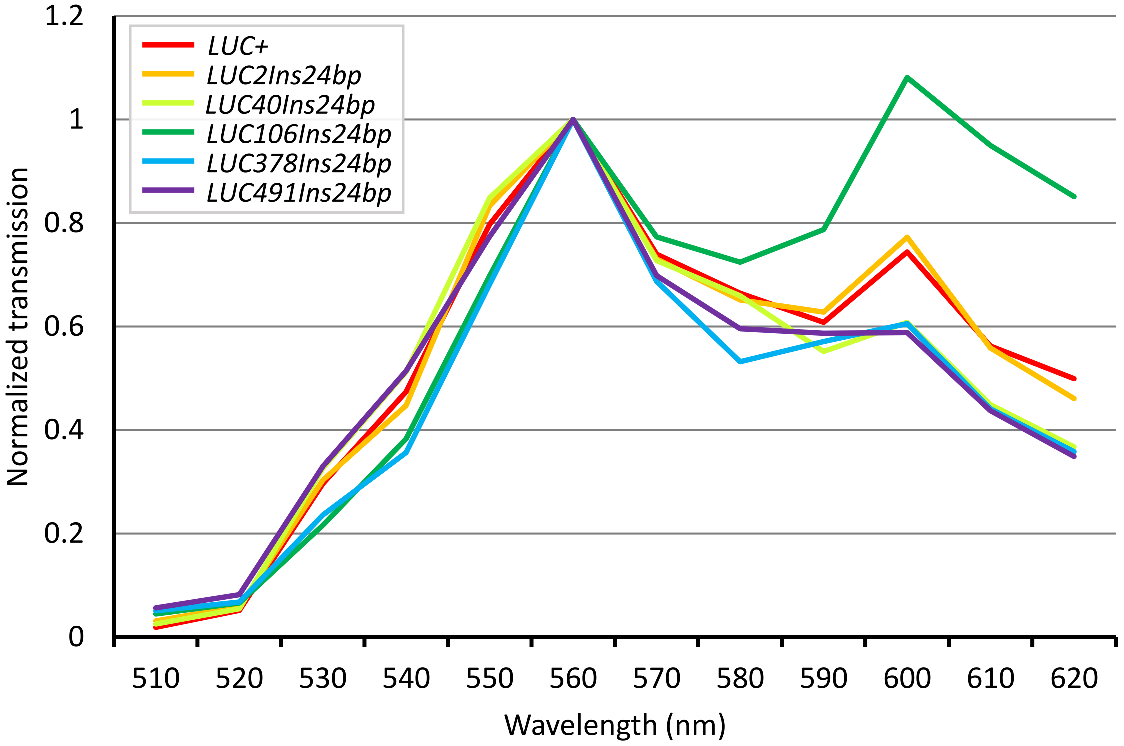

Bioluminescence spectra of *LUCX$_{AA}$Ins24bp*. The transmitted bioluminescence intensities from tested constructs were normalized to that at 560 nm were plotted. Bioluminescence was filtered with a series of band-pass filters and quantified [25].

Supplementary Fig. S3

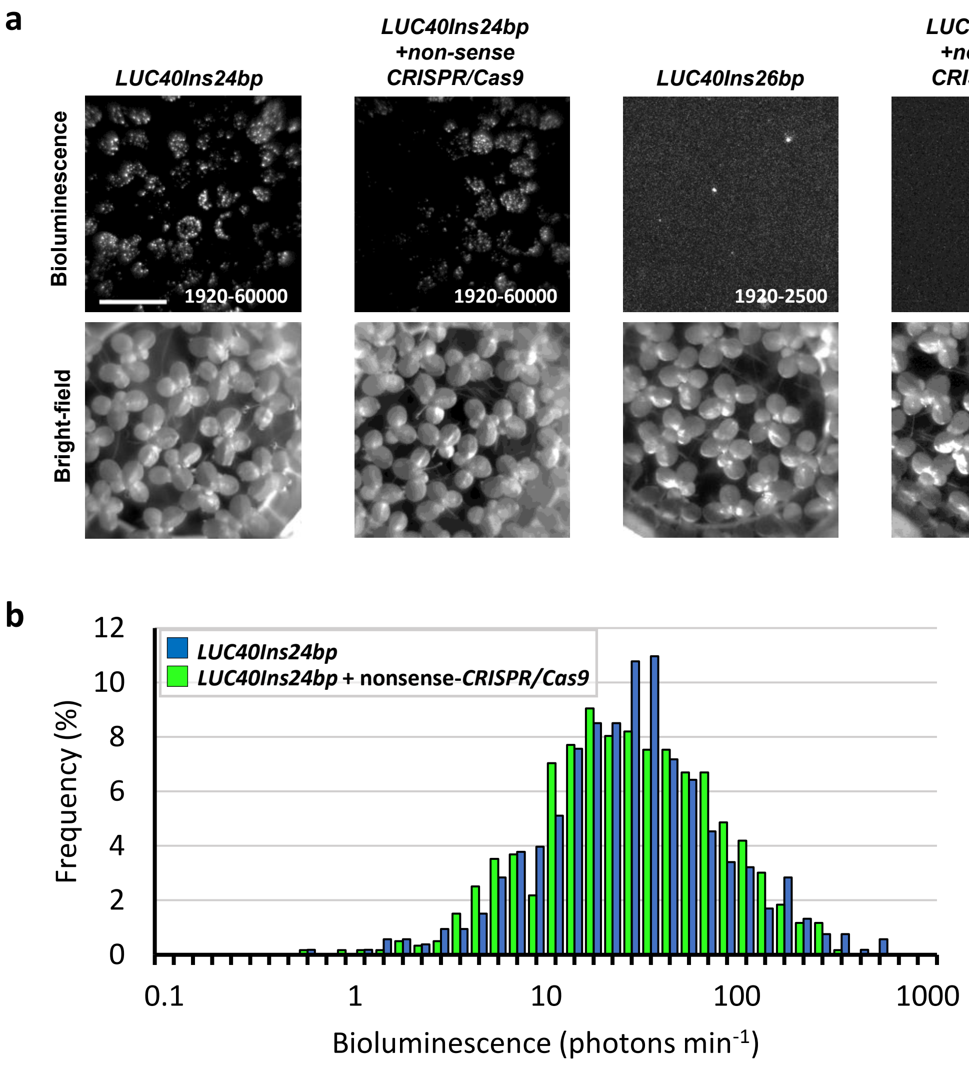

Bioluminescence in duckweed cells co-transfected with *LUC40Ins24bp* or *LUC40Ins26bp* and nonsense-*CRISPR/Cas9* constructs. (**a**) Bioluminescence (top) and bright-field (bottom) images of duckweed plants transfected with a reporter construct or both a reporter and nonsense-*CRISPR/Cas9* constructs (indicated above each set of images). Signal ranges are indicated in each bioluminescence image. Exposure time: 60 s. Bar: 10 mm. (**b**) Frequency distribution (%) of bioluminescence intensities in cells (co-)transfected with *CaMV35S::LUC40Ins24bp* (blue bars; n = 529) or *CaMV35S::LUC40Ins24bp* and nonsense-*CRISPR/Cas9* constructs (green bars; n = 597). The mean bioluminescence intensity of *CaMV35S::LUC40Ins24bp* and nonsense-*CRISPR/Cas9* constructs is compared to that of *CaMV35S::LUC40Ins24bp* (p = 0.00033 by Welch's two-tailed t-test). Twenty-five duckweed colonies were observed in one experiment. The images (**a**) and bioluminescence distribution (**b**) of *CaMV35S::LUC40Ins24bp* and *CaMV35S::LUC40Ins26bp* are the same as Fig. 3a and Fig. 3c, respectively.

Supplementary Fig. S4

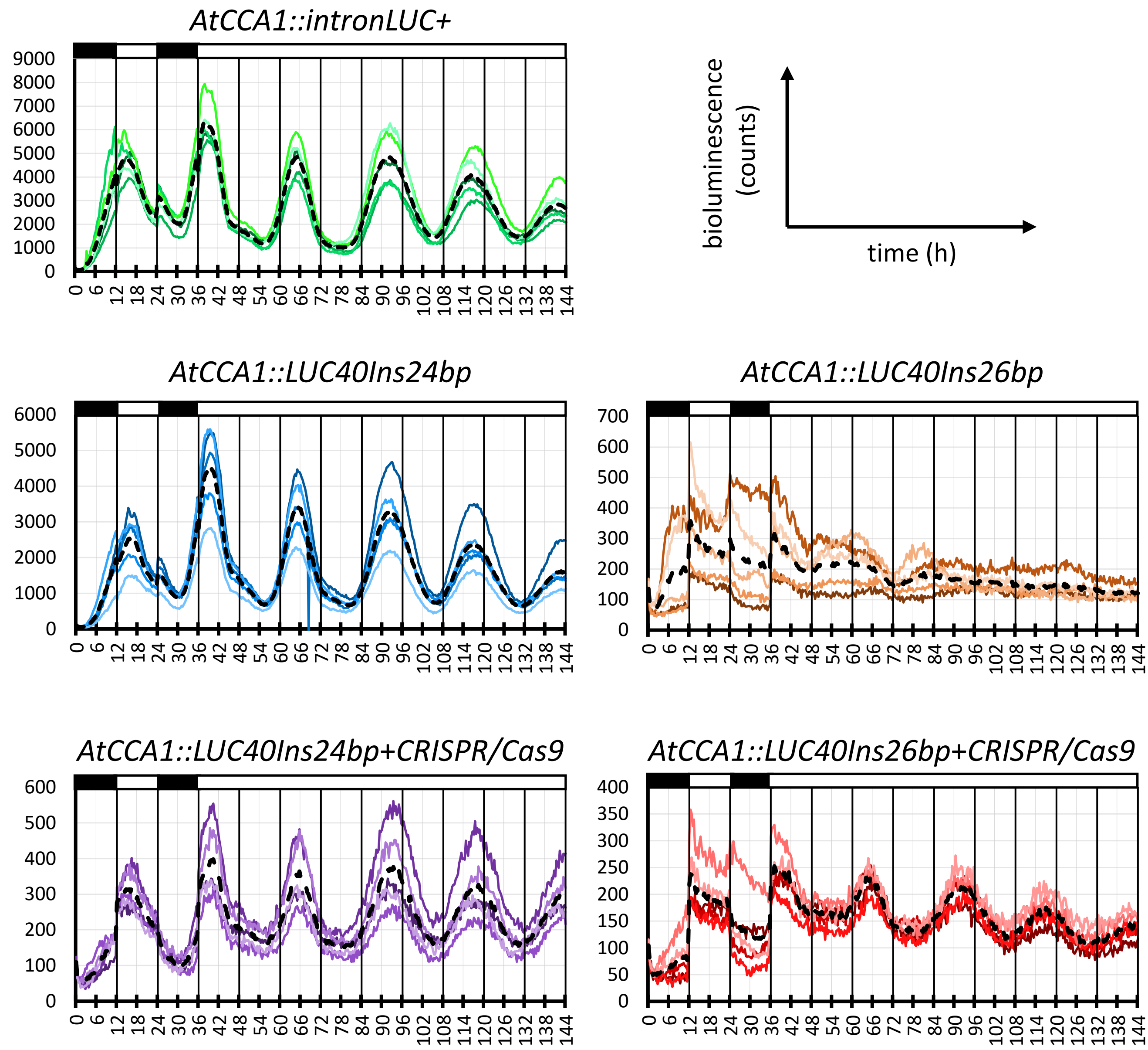

Effects of the *CRISPR/Cas9* constructs on bioluminescence rhythms of duckweed plants transiently transfected with *AtCCA1::LUC40Ins24bp* and *AtCCA1::LUC40Ins26bp* reporters. Each graph represents a time series of the bioluminescence intensities observed in duckweed plants transfected with a reporter construct and the *CRISPR/Cas9* construct (indicated above each graph). Black dotted lines represent the mean bioluminescence intensities of five samples. As previously described, bioluminescence was measured using an automatic luminescence monitoring system with a photomultiplier tube [48]. The *intronLUC+* gene has an intron within the *LUC+* coding region [48]. Black and white boxes indicate dark and light conditions, respectively.

Supplementary Fig. S5

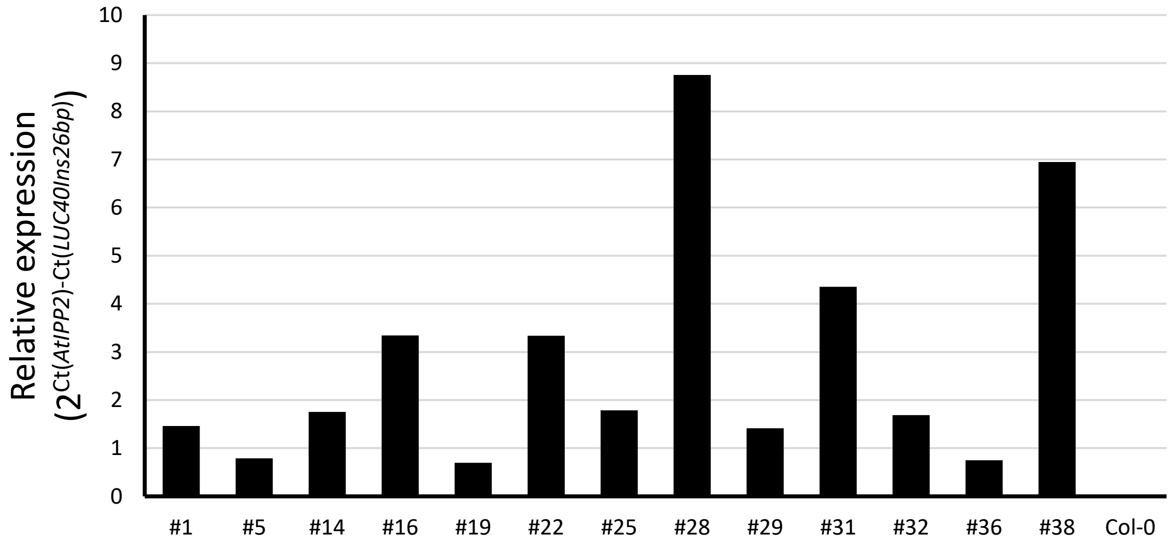

mRNA expression levels of *LUC40Ins26bp* in T*2* transgenic *Arabidopsis* plants carrying *CaMV35S::LUC40Ins26bp*. *LUC40Ins26bp* mRNA levels, quantified by qPCR, are shown for 13 independent transgenic lines and *Col-0*. Based on the segregation of hygromycin B resistance, these lines were estimated as single-locus transformants.

Supplementary Fig. S6

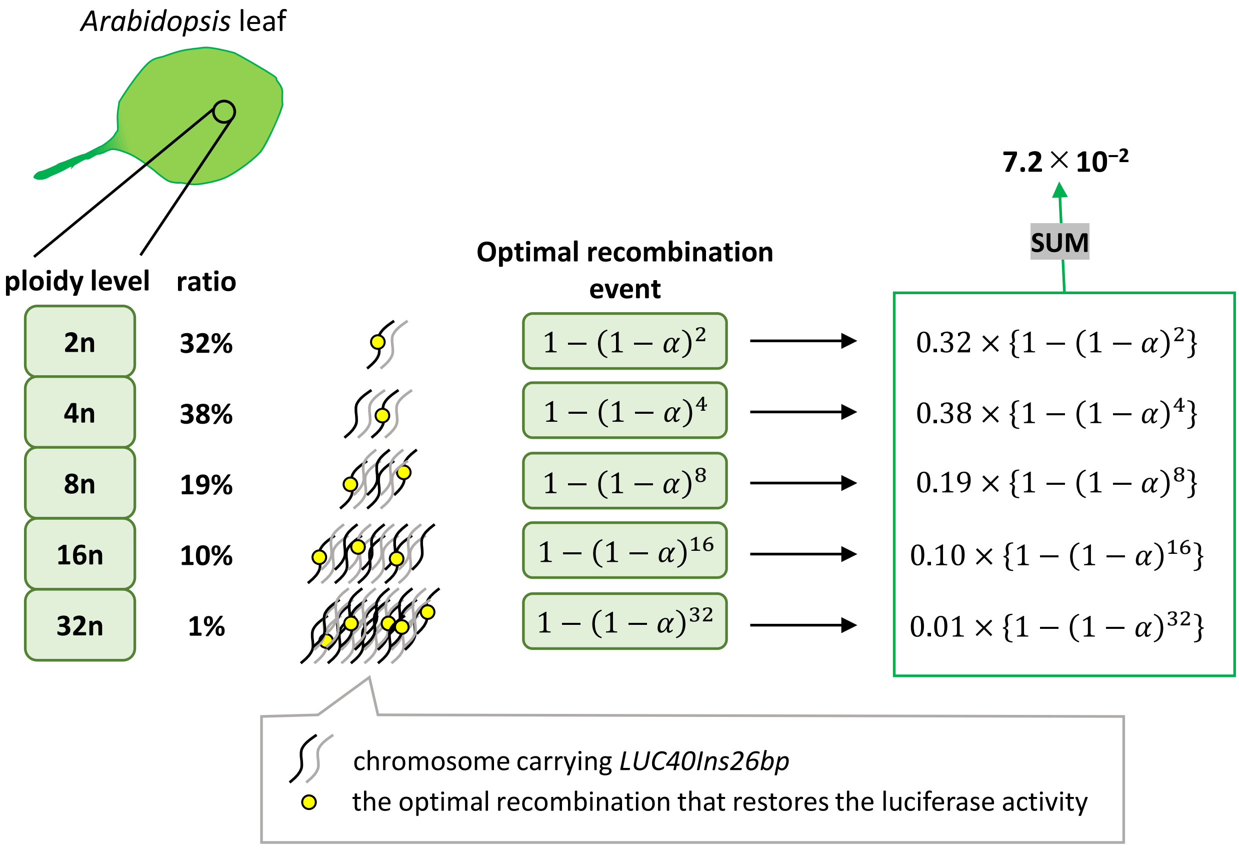

Relationship between the ploidy profile of *Arabidopsis* leaf cells and the ratio of bioluminescent spots. The indicated ploidy levels represent the ploidy profile of *Arabidopsis* leaf epidermal cells (2n:4n:8n:16n:32n = 0.32:0.38:0.19:0.10:0.01) reported by Kawade and Tsukaya, 2017 [49]. The probability of the optimal recombination was defined as α. The probability of restoring cellular bioluminescence by the optimal recombination was expressed as follows;

$$1-(1-\alpha)^{(\text{ploidy level})}.$$

The ratio of bioluminescence-restored cells to transfected cells (7.2×10^{-2}; Fig. 4) was simulated as the sum of the probabilities with a value of $\alpha = 1.37 \times 10^{-2}$.

Supplementary Fig. S7

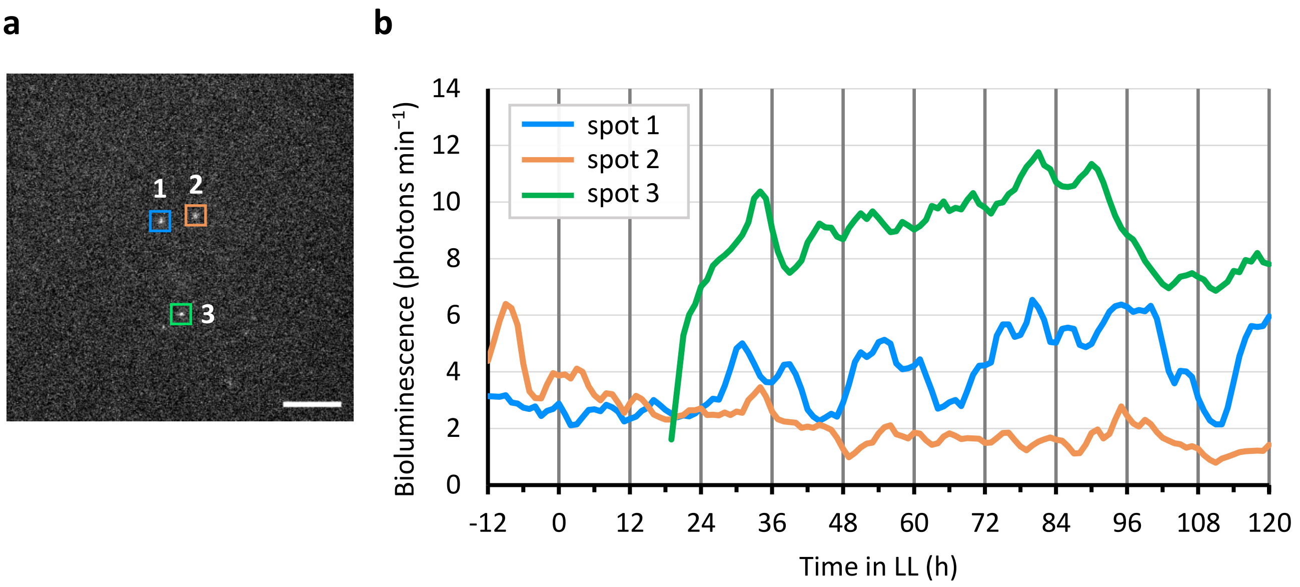

Cellular bioluminescence traces of restored-*LUC40Ins26bp* in transgenic plants (line *LUC40Ins26bp* #38-2). (**a**) Snapshot of bioluminescence at 28 h in constant light. Each colored square represents one bioluminescent spot of interest. Spot 3 belongs to a different leaf from that bearing spots 1 and 2. Bar: 2 mm. (**b**) Cellular bioluminescence traces of the three bioluminescent spots shown in (**a**). Quantified intensities (3-h moving average) are plotted for each spot. Time schedule was the same as Fig. 6.

Supplementary Fig. S8

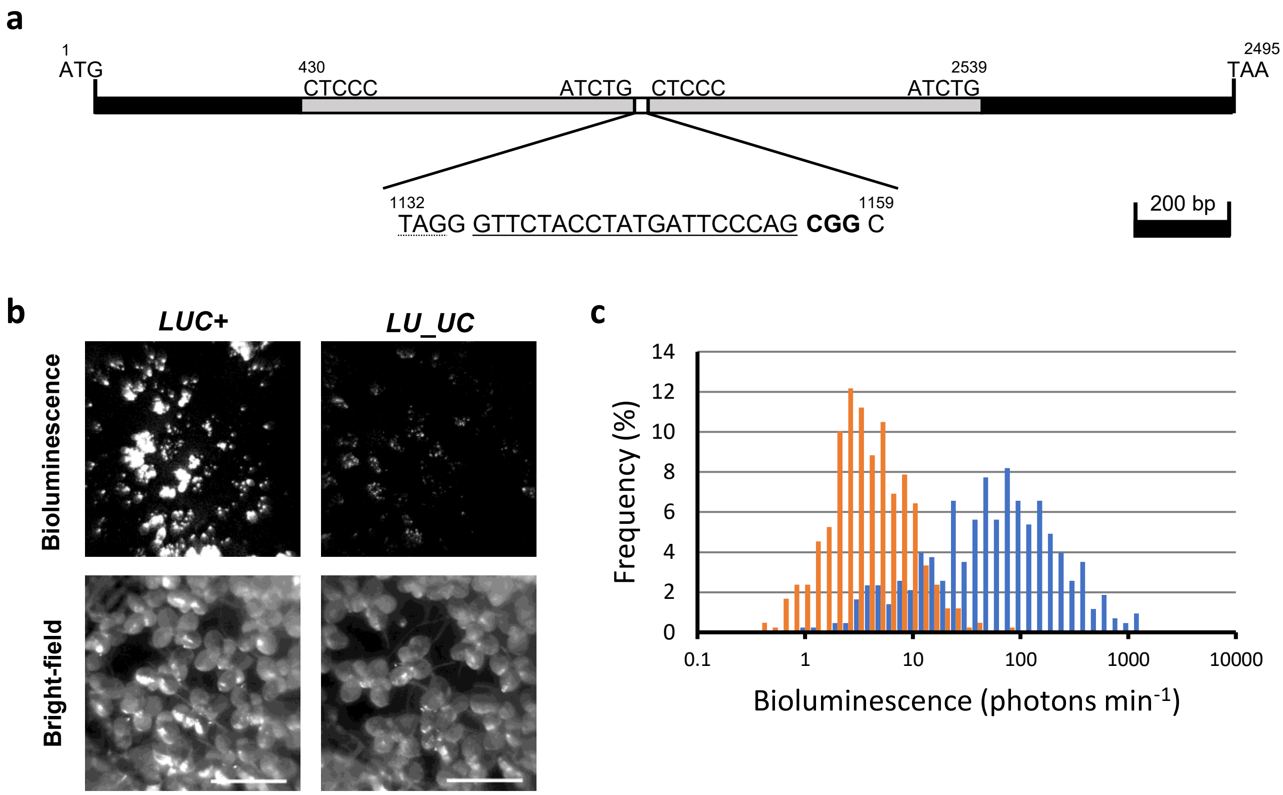

***LU_UC* construct and its bioluminescence properties.** (**a**) Map of the *LU_UC* gene. The region encompassing from 430 bp to 1131 bp of the *LUC+* gene is duplicated. A 28-bp fragment (1132–1159 bp) was inserted between these duplicated sequences. The *sgRNA* target sequence for CRISPR/Cas9 is underlined, whereas the PAM sequence CGG is indicated in bolds. TAG, a stop codon, is underlined with a dotted line. (**b**) Bioluminescence (top) and bright-field (bottom) images of duckweed plants transfected with *CaMV35S::LUC+* (left) and *CaMV35S::LU_UC* (right). The signal range for each bioluminescence image was fixed to 1920–5000. Exposure time: 30 s. Bars: 10 mm. (**c**) Frequency distribution (%) of cellular bioluminescence intensities for *CaMV35S::LUC+* (blue bars, n = 427) and *CaMV35S::LU_UC* (orange bars, n = 419). The mean bioluminescence intensity of *CaMV35S::LU_UC* is compared to that of *CaMV35S::LUC+* ($p = 1.8 \times 10^{-152}$ by Welch's two-tailed t-test). Twenty-five duckweed colonies were observed in one experiment.

Supplementary Fig. S9

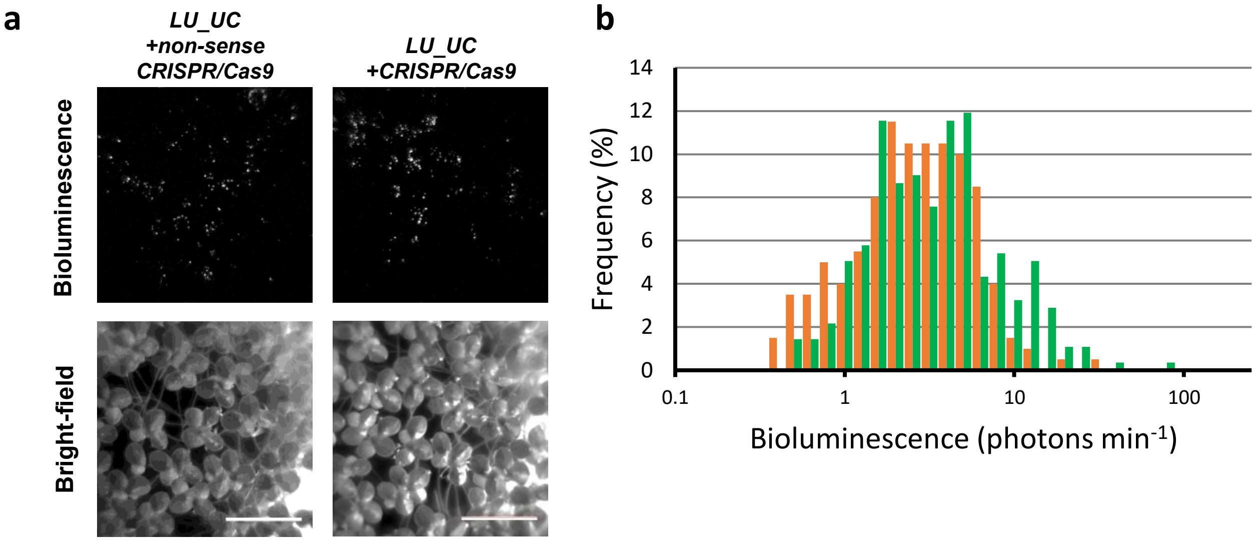

Comparison of cellular bioluminescence intensities between duckweed cells co-transfected with *LU_UC* and nonsense-*CRISPR/Cas9* or *CRISPR/Cas9* constructs. (**a**) Bioluminescence (top) and bright-field (bottom) images of duckweed plants co-transfected with *CaMV35S::LU_UC* and nonsense-*CRISPR/Cas9* constructs (left), and *CaMV35S::LU_UC* and *CRISPR/Cas9* constructs (right). The signal range for each bioluminescence image was fixed to 1920–5000. Exposure time: 60 s. Bars: 10 mm. (**b**) Frequency distribution (%) of cellular bioluminescence intensities for *CaMV35S::LU_UC* and nonsense-*CRISPR/Cas9* constructs (orange bars, n = 200), and *CaMV35S::LU_UC* and *CRISPR/Cas9* constructs (green bars, n = 277). The mean bioluminescence intensity of *CaMV35S::LU_UC* and nonsense-*CRISPR/Cas9* constructs is compared to that of *CaMV35S::LU_UC* and *CRISPR/Cas9* constructs (p = 0.00064 by Welch's two-tailed t-test). Twenty-five duckweed colonies were observed in one experiment.

Supplementary Fig. S10

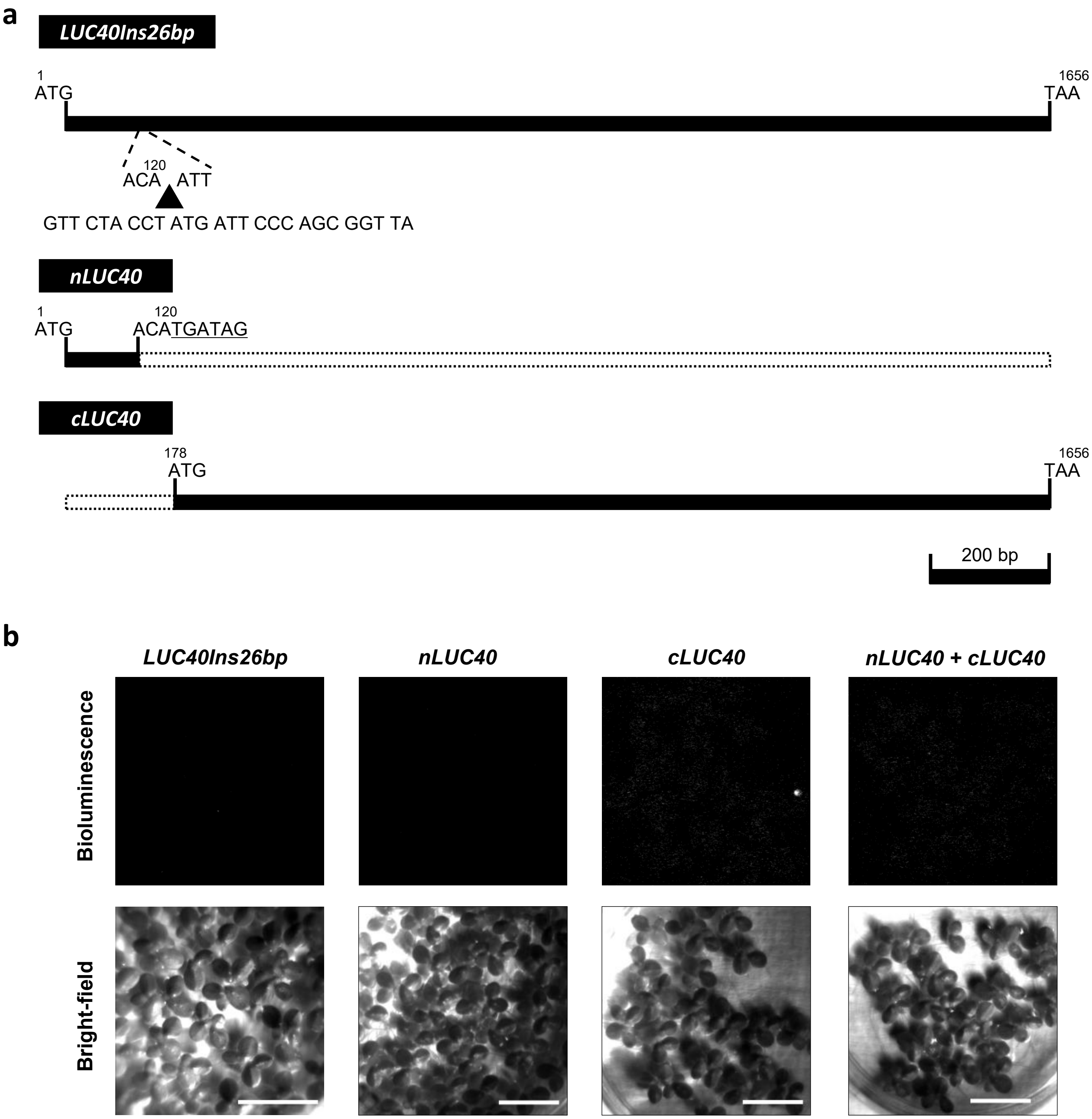

Split *LUC40Ins24bp* constructs (*nLUC40* and *cLUC40*) and bioluminescence properties. (**a**) *LUC40Ins26bp* (Fig. 1), *nLUC40*, and *cLUC40* gene maps. The *nLUC40* sequence has two stop codons at its end (underlined). White dotted boxes indicate deleted regions. (**b**) Bioluminescence (top) and bright-field (bottom) images of duckweed plants transfected with *CaMV35S::LUC40Ins26bp, CaMV35S::nLUC40, CaMV35S::cLUC40*, and *CaMV35S::nLUC40* + *CaMV35S::cLUC40* (from left to right). Exposure time: 90 s. The signal range for the bioluminescence images was fixed to 1920–2200. Bar: 10 mm.

Supplementary Fig. S11

CCCCCATCACAACTTTGTATAGAAAAGTTGGCTCCGAATTCGCCCTTACCCACAGATGGTTAGAGAGGCTTACGCAGCAGGTCTCATCAA
GACGATCTACCCGAGCAATAATCTCCAGGAAATCAAATACCTTCCCAAGAAGGTTAAAGATGCAGTCAAAAGATTCAGGACTAACTGCAT
CAAGAACACAGAGAAAGATATATTTCTCAAGATCAGAAGTACTATTCCAGTATGGACGATTCAAGGCTTGCTTCACAAACCAAGGCAAGT
AATAGAGATTGGAGTCTCTAAAAAGGTAGTTCCCACTGAATCAAAGGCCATGGAGTCAAAGATTCAAATAGAGGACCTAACAGAACTCGC
CGTAAAGACTGGCGAACAGTTCATACAGAGTCTCTTACGACTCAATGACAAGAAGAAAATCTTCGTCAACATGGTGGAGCACGACACACT
TGTCTACTCCAAAAATATCAAAGATACAGTCTCAGAAGACCAAAGGGCAATTGAGACTTTTCAACAAAGGGTAATATCCGGAAACCTCCT
CGGATTCCATTGCCCAGCTATCTGTCACTTTATTGTGAAGATAGTGGAAAAGGAAGGTGGCTCCTACAAATGCCATCATTGCGATAAAGG
AAAGGCCATCGTTGAAGATGCCTCTGCCGACAGTGGTCCCAAAGATGGACCCCCACCCACGAGGAGCATCGTGGAAAAAGAAGACGTTCC
AACCACGTCTTCAAAGCAAGTGGATTGATGTGATATCTCCACTGACGTAAGGGATGACGCACAATCCCACTATCCTTCGCAAGACCCTTC
CTCTATATAAGGAAGTTCATTTCATTTGGAGAGAACACGAAGGGCGAATTCGACCCAAGTTTGTACAAAAAAGCAGGCTCCGCGGCCGCC
ATGGTCACCGACGCCAAAAACATAAAGAAAGGCCCGGCGCCATTCTATCCGCTGGAAGATGGAACCGCTGGAGAGCAACTGCATAAGGCT
ATGAAGAGATACGCCCTGGTTCCTGGAACAGTTCTACCTATGATTCCCAGCGGTTAATTGCTTTTACAGATGCACATATCGAGGTGGACA
TCACTTACGCTGAGTACTTCGAAATGTCCGTTCGGTTGGCAGAAGCTATGAAACGATATGGGCTGAATACAAATCACAGAATCGTCGTAT
GCAGTGAAAACTCTCTTCAATTCTTTATGCCGGTGTTGGGCGCGTTATTTATCGGAGTTGCAGTTGCGCCCGCGAACGACATTTATAATG
AACGTGAATTGCTCAACAGTATGGGCATTTCGCAGCCTACCGTGGTGTTCGTTTCCAAAAAGGGGTTGCAAAAAATTTTGAACGTGCAAA
AAAAGCTCCCAATCATCCAAAAAATTATTATCATGGATTCTAAAACGGATTACCAGGGATTTCAGTCGATGTACACGTTCGTCACATCTC
ATCTACCTCCCGGTTTTAATGAATACGATTTTGTGCCAGAGTCCTTCGATAGGGACAAGACAATTGCACTGATCATGAACTCCTCTGGAT
CTACTGGTCTGCCTAAAGGTGTCGCTCTGCCTCATAGAACTGCCTGCGTGAGATTCTCGCATGCCAGAGATCCTATTTTTGGCAATCAAA
TCATTCCGGATACTGCGATTTTAAGTGTTGTTCCATTCCATCACGGTTTTGGAATGTTTACTACACTCGGATATTTGATATGTGGATTTC
GAGTCGTCTTAATGTATAGATTTGAAGAAGAGCTGTTTCTGAGGAGCCTTCAGGATTACAAGATTCAAAGTGCGCTGCTGGTGCCAACCC
TATTCTCCTTCTTCGCCAAAAGCACTCTGATTGACAAATACGATTTATCTAATTTACACGAAATTGCTTCTGGTGGCGCTCCCCTCTCTA
AGGAAGTCGGGGAAGCGGTTGCCAAGAGGTTCCATCTGCCAGGTATCAGGCAAGGATATGGGCTCACTGAGACTACATCAGCTATTCTGA
TTACACCCGAGGGGGATGATAAACCGGGCGCGGTCGGTAAAGTTGTTCCATTTTTTGAAGCGAAGGTTGTGGATCTGGATACCGGGAAAA
CGCTGGGCGTTAATCAAAGAGGCGAACTGTGTGTGAGAGGTCCTATGATTATGTCCGGTTATGTAAACAATCCGGAAGCGACCAACGCCT
TGATTGACAAGGATGGATGGCTACATTCTGGAGACATAGCTTACTGGGACGAAGACGAACACTTCTTCATCGTTGACCGCCTGAAGTCTC
TGATTAAGTACAAAGGCTATCAGGTGGCTCCCGCTGAATTGGAATCCATCTTGCTCCAACACCCCAACATCTTCGACGCAGGTGTCGCAG
GTCTTCCCGACGATGACGCCGGTGAACTTCCCGCCGCCGTTGTTGTTTTGGAGCACGGAAAGACGATGACGGAAAAAGAGATCGTGGATT
ACGTCGCCAGTCAAGTAACAACCGCGAAAAAGTTGCGCGGAGGAGTTGTGTTTGTGGACGAAGTACCGAAAGGTCTTACCGGAAAACTCG
ACGCAAGAAAAATCAGAGAGATCCTCATAAAGGCCAAGAAGGGCGGAAAGATCGCCGTGTAAGGCGCGCCGACCCAGCTTTCTTGTACAA
AGTGGTTGATAACAGCGCTTAGAGCTCGAATTTCCCCGATCGTTCAAACATTTGGCAATAAAGTTTCTTAAGATTGAATCCTGTTGCCGG
TCTTGCGATGATTATCATATAATTTCTGTTGAATTACGTTAAGCATGTAATAATTAACATGTAATGCATGACGTTATTTATGAGATGGGT
TTTTATGATTAGAGTCCCGCAATTATACATTTAATACGCGATAGAAAACAAAATATAGCGCGCAAACTAGGATAAATTATCGCGCGCGGT
GTCATCTATGTTACTAGATCGGGAATTGGTTCCGGAACCAATTCGTAATCATGTCATAGCTGTTTCCTGTGTGAAATTGTTATCCGCTCA

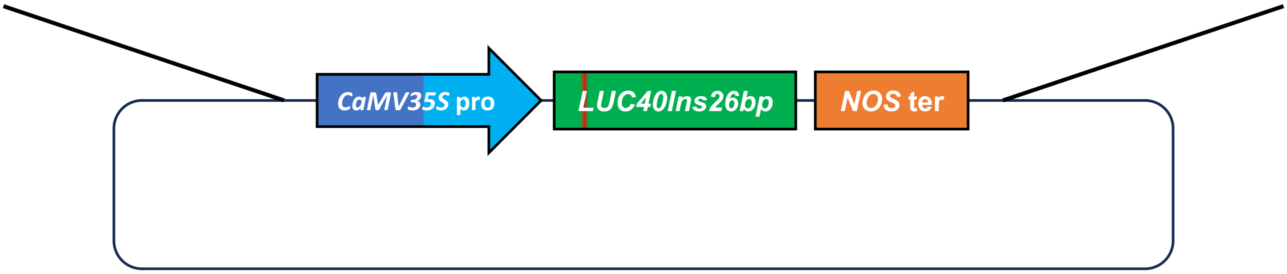

Sequence information of *pR4GWB501_CaMV35S::LUC40Ins26bp*. Deep blue and light blue indicate the dual *CaMV35S* promoter. Green indicates the original *LUC*+ sequence, and red indicates the 26-bp insertion including the *sgRNA* target sequence (under bar) and the PAM sequence (yellow highlight). Orange indicates *NOS* terminator. One row contains 90 characters.
